# Evaluation of PermaNet 3.0 a deltamethrin-PBO combination net against *Anopheles gambiae *and pyrethroid resistant *Culex quinquefasciatus *mosquitoes: an experimental hut trial in Tanzania

**DOI:** 10.1186/1475-2875-9-21

**Published:** 2010-01-19

**Authors:** Patrick Tungu, Stephen Magesa, Caroline Maxwell, Robert Malima, Dennis Masue, Wema Sudi, Joseph Myamba, Olivier Pigeon, Mark Rowland

**Affiliations:** 1 Amani Medical Research Centre, National Institute for Medical Research, PO Box 81, Muheza, Tanzania; 2 Pan-African Malaria Vector Research Consortium, Tanzania; 3 Pesticides Research Department, Walloon Agricultural Research Centre, 11 Rue du Bordia, B-5030 Gembloux, Belgium; 4 Department of Infectious Diseases, London School of Hygiene and Tropical Medicine, WC1E 7HT, London, UK

## Abstract

**Background:**

Combination mosquito nets incorporating two unrelated insecticides or insecticide plus synergist are designed to control insecticide resistant mosquitoes. PermaNet 3.0 is a long-lasting combination net incorporating deltamethrin on the side panels and a mixture of deltamethrin and synergist piperonyl butoxide (PBO) on the top panel. PBO is an inhibitor of mixed function oxidases implicated in pyrethroid resistance.

**Method:**

An experimental hut trial comparing PermaNet 3.0, PermaNet 2.0 and a conventional deltamethrin-treated net was conducted in NE Tanzania using standard WHOPES procedures. The PermaNet arms included unwashed nets and nets washed 20 times. PermaNet 2.0 is a long-lasting insecticidal net incorporating deltamethrin as a single active.

**Results:**

Against pyrethroid susceptible Anopheles gambiae the unwashed PermaNet 3.0 showed no difference to unwashed PermaNet 2.0 in terms of mortality (95% killed), but showed differences in blood-feeding rate (3% blood-fed with PermaNet 3.0 versus 10% with PermaNet 2.0). After 20 washes the two products showed no difference in feeding rate (10% with 3.0 and 9% with 2.0) but showed small differences in mortality (95% with 3.0 and 87% with 2.0). Against pyrethroid resistant Culex quinquefasciatus, mediated by elevated oxidase and kdr mechanisms, the unwashed PermaNet 3.0 killed 48% and PermaNet 2.0 killed 32% but after 20 washes there was no significant difference in mortality between the two products (32% killed by 3.0 and 30% by 2.0). For protecting against Culex PermaNet 3.0 showed no difference to PermaNet 2.0 when either unwashed or after 20 washes; both products were highly protective against biting. Laboratory tunnel bioassays confirmed the loss of biological activity of the PBO/deltamethrin-treated panel after washing.

**Conclusion:**

Both PermaNet products were highly effective against susceptible Anopheles gambiae. As a long-lasting net to control or protect against pyrethroid resistant mosquitoes PermaNet 3.0 showed limited improvement over PermaNet 2.0 against Culex quinquefasciatus.

## Background

The development of insecticide resistance is probably the biggest threat to capacity to control malaria vectors or sustain any drive towards malaria elimination. The chemical agents that make malaria vector control feasible are the pyrethroids. The best tools for delivering pyrethroids are long-lasting insecticidal nets (LLIN) and indoor residual spraying (IRS) [1]. Recent trends confirm that the scale up of these two tools is making inroads into the malaria burden in many African countries [2-6]. This has stimulated new discussion about malaria elimination which a few years ago seemed inconceivable [7-11]. But coinciding with the increased coverage of LLIN and IRS is the development and spread of resistant mosquitoes that may ultimately undermine the effectiveness of the two tools [12-16]. For elimination to remain a realistic prospect, it is essential to preserve the pyrethroids for as long as possible because no other insecticide class can match the pyrethroids for effectiveness, safety, cost per unit dose, acceptability or suitability for LLIN and IRS [17,18].

Resistance management has a theoretical foundation in population genetics that goes back three decades [19-21]. Simulation modeling has shown that the most promising way to delay the selection of resistance is to apply mixtures of unrelated insecticides [22-25]. The idea behind mixtures is that insects which develop resistance to one insecticide should be killed by the second insecticide provided they are not resistant to both and a proportion of each generation escapes exposure altogether. When resistance is present at low frequency - such as when it first evolves - double resistance will be rare and selection of each type of resistance should be delayed or prevented.

The same principle has been adopted in the strategy to preserve anti-malarial drug efficacy known as combination therapy [1,26]. Adoption of combination therapy on the Thai-Burmese border has prevented or delayed the selection of drug resistance when in the preceding decade chloroquine, SP and mefloquine monotherapy were each rendered redundant by sequential evolution of resistance [27]. It is time a similar strategy was adopted for preserving insecticides for malaria vector control.

Alternative insecticides to pyrethroids have been tested on nets for effect against wild, pyrethroid resistant mosquito populations but only under limited experimental conditions in the field [28-31]. Most alternatives lack the excito-repellency of pyrethroids, a characteristic important for reducing biting rates or providing personal protection to users of insecticide-treated nets (ITNs). This limitation is the main reason for combining the alternative insecticide with a pyrethroid that is capable of adding repellency to the product. Combinations can be applied either as a mixture of the two insecticides or as a two-in-one (mosaic) format, in which the pyrethroid is restricted to the sides and the alternative insecticide to the top of the net [29,31]. For the two-in-one net to work as a resistance management tactic mosquitoes should contact both the top and sides so that any pyrethroid resistant mosquito that survives contact with the pyrethroid stands a high chance of being killed by the alternative insecticide. There is indirect evidence that host-seeking mosquitoes of the *An. gambiae *complex do in fact contact the top [32] possibly in response to odour plumes or concentration gradients, and this gives the two-in-one concept a degree of credibility.

Rather than use a non-pyrethroid insecticide to overcome resistance, an equally valid approach is to deploy a chemical synergist on the fibres. Synergists overcome resistance by inhibiting the enzymes responsible for certain types of resistance. Resistance to pyrethroids in Anopheline mosquitoes appears to be caused by two primary mechanisms: a target site insensitivity mechanism known as *kdr *and a metabolic mechanism caused by mixed function oxidases (MFOs). MFOs are responsible for the pyrethroid resistance that evolved in *Anopheles funestus *and which led to the failure of IRS campaigns in South Africa [12,33]. It appears that MFOs may also act in consort with *kdr *to create a pyrethroid resistance, that is causing control failure of *Anopheles gambiae *M form in parts of West Africa [13,14,34]. Both MFOs and *kdr *together are responsible for pyrethroid resistance in *Culex quinquefasciatus *[35,36]. One type of synergist capable of inhibiting MFOs is piperonyl butoxide (PBO). PBO is commonly used in commercial aerosols for potentiating pyrethroid activity against flying or domestic insect pests [18].

PBO has potential to combat the growing problem of pyrethroid resistance in *An. gambiae *and other vector species. PermaNet 3.0 is a long-lasting insecticidal net developed by Vestergaard Frandsen in which the PBO together with the pyrethroid deltamethin are incorporated into the polyethylene fibres on the roof panel of the net. The sides of PermaNet 3.0 are made of polyester and coated with a long-lasting formulation of deltamethrin similar to the pyrethroid-based LLIN, PermaNet 2.0 but with a strengthened lower part. By restricting PBO to the roof of the net the concept of PermaNet 3.0 is to have the insect make contact with the synergist on the roof, mediated by the odour plume, before making further contact with pyrethroid on the sides during exploration.

PermaNet 3.0 was submitted by Vestergaard Frandsen to the WHO Pesticide Evaluation Scheme (WHOPES) for formal evaluation. The current paper reports upon a WHOPES-sponsored experimental hut trial conducted against wild, free flying *An. gambiae *and *Cx quinquefasciatus *in Muheza, Tanzania, together with supporting laboratory data and chemical analysis.

## Methods

### Long-lasting insecticidal nets

PermaNet 3.0 LN (Vestergaard Frandsen SA, Denmark) is a LLIN consisting of a top panel made of monofilament polyethylene (100 denier) fabric incorporating deltamethrin at 4 g/kg (approx. 180 mg/m^2^) and piperonyl butoxide at 25 g/kg (approx. 1.1 g/m^2^), plus side panels made of multifilament polyester (75 denier) fabric with a strengthened border treated with deltamethrin at 2.8 g/kg (approx. 118 mg/m^2^).

PermaNet 2.0 LN (Vestergaard Frandsen SA, Denmark) is a LLIN made of multifilament polyester (75-100 denier) fabric, factory treated with a wash-resistant formulation of deltamethrin at 1.8 g/kg (for 75 denier) (approx. 62 mg/m^2^).

The conventionally treated net (CTN) is a multifilament polyester (100 denier) fabric treated with deltamethrin (K-Othrine SC, Bayer) at 25 mg/m^2^; the net was treated by hand, on site, in an aqueous solution of the formulation.

The size of the PermaNet 2.0 and standard nets was 130 cm wide, 190 cm long, 150 cm high. PermaNet 3.0 nets measured 120 cm wide, 190 cm long, 150 cm high. Hence the top panel of PermaNet 3.0 containing the PBO plus deltamethrin constituted 19.7% of the overall surface area whereas the remaining 80.3% on the sides contained only deltamethrin as an active ingredient.

### Mosquito strains

*Anopheles gambiae *sensu stricto Kisumu, a laboratory insecticide susceptible strain, originally from Kenya.

*Culex quinquefasciatus *TPRI, a laboratory insecticide susceptible strain, maintained by the Tropical Pesticide Research Institute, Tanzania.

*Culex quinquefasciatus *Masimbani, a multiple resistant strain from northeast Tanzania, containing elevated oxidase and *kdr *pyrethroid resistance mechanisms. In WHO resistance tests the strain showed survival after one hour exposure to test papers of permethrin (47% survival) deltamethrin (48%), DDT (58%), malathion (27%) and propoxur (46%).

### Exploratory bioassay tests on PermaNet 3.0, PermaNet 2.0 and CTN washed up to 20 times

#### Cone bioassays

Three min exposure bioassay tests were carried out using *An. gambiae *Kisumu (pyrethroid susceptible) on PermaNet 3.0, PermaNet 2.0 and the CTN after 0, 10 and 20 washes. Similarly *Cx. quinquefasciatus *Masimbani (pyrethroid resistant) was exposed in 3 min bioassay tests to netting taken from the roof and sides of the PermaNet 3.0 after 0, 10 and 20 wash intervals. Washing was carried out on entire nets using the WHO Phase II washing protocol [37]. Bioassays were conducted with WHO cones on netting samples, using five 3-5 day old mosquitoes per replicate and 10 replicate tests per treatment [37,38]. After the 3 min exposures mosquitoes were aspirated from the cones and held in paper cups and provided with 10% glucose solution. Mortality was recorded after 24 h.

#### Tunnel tests

The tunnel tests were carried out on samples of PermaNet 3.0 netting cut from the roof and lower sides of the net after 0, 10 and 20 washes using the Phase II washing procedure [37]. The tests were conducted using laboratory-reared *An. gambiae *Kisumu (insecticide susceptible), *Cx. quinquefasciatus *TPRI (insecticide susceptible) and *Cx. quinquefasciatus *Masimbani (pyrethroid resistant). Tunnel tests were replicated three times.

The tunnel test apparatus is comprised of a glass cuboid tube, 25 cm high, 21 cm wide, 60 cm long, divided into two chambers by a transverse netting insert fitted onto a frame which slots across the tunnel. Nine 1 cm diameter holes were cut into the netting to allow passage of mosquitoes. In the bait chamber, a guinea pig was housed unconstrained in a wire meshed cage and in the other chamber 100 unfed female mosquitoes 3-5 days old were released at dusk and left overnight in the dark as per WHO guidelines [37,38]. The following morning the numbers of mosquitoes found live or dead, fed or unfed in each compartment were recorded. Live mosquitoes were transferred to paper cups and supplied with a pad of cotton wool soaked in 10% glucose solution. Delayed mortality was observed after 24 h.

### Experimental hut trial

#### Determination of the point of 'insecticide exhaustion'

A polyester net conventionally treated with deltamethrin at dosage 25 mg/m^2 ^was washed until just before 'insecticide exhaustion' as defined by WHO [37]. The conventionally treated net (CTN) treatment serves as a positive control to judge PermaNet 3.0 performance against. The point of exhaustion is the point at which the CTN showed less than 80% mortality or 95% knock down in WHO cone bioassays conducted after each wash. The standardized WHO washing protocol requires the net to be stirred in 10 litres of soap solution (2 g/litre of 'Savon de Marseille') for 6 min, during a 10 min washing cycle at ambient temperature. Nets were rinsed and dried and left for one day between washes. Determination of the 'point of exhaustion' was carried out by exposing unfed *An. gambiae *Kisumu in 10 replicates of 5 mosquitoes per replicate at each wash interval on the five panels of each net. Exposure was for 3 min and mortality was scored 24 h later.

#### Study area and hut design

The six veranda trap huts were situated at Zeneti village, Muheza district, NE Tanzania (5°13'S and 38°39'E). They were constructed according to a design first described by Smith [39], but built on concrete plinths surrounded by water-filled moats to deter entry of scavenging ants. The brick walls were plastered with mud on the inside, the roofs made of corrugated iron, the wooden ceilings lined with Hessian sackcloth, with open eaves and veranda traps and window traps on each side of the hut. The veranda traps on two opposing sides were closed to capture any mosquitoes that exit via the eaves. The two verandas on the other two sides were left open so mosquitoes can enter the huts through the gaps under the eaves. Each night's collection inside the two screened veranda traps was multiplied by two and added to the room and window trap collections; the multiplication was to adjust for the unrecorded escapes through the two verandas which were left unscreened to allow routes for entry of wild mosquitoes via the gaps under the eaves. At the end of each rotation the north and south verandas were closed and east and west sides opened, or *vice versa*, to compensate for possible selective exiting in one compass direction.

*Anopheles gambiae *s.s., *An. funestus *and *Cx. quinquefasciatus *are the predominant mosquito species in the area. The *An. gambiae *and *An. funestus *are susceptible to pyrethroids; *Cx. quinquefasciatus *is resistant to pyrethroids, mediated by enhanced oxidase and site insensitivity mechanisms [[36], Malima & Rowland, unpublished data]. The timing of the trial was set during a period when both *An. gambiae *and *Cx. quinquefasciatus *were abundant. The wild adult mosquitoes were characterized for resistance by testing with deltamethin 0.05% papers in WHO test kits.

#### Study design

The following six treatment arms were compared:

1. Unwashed PermaNet 3.0

2. PermaNet 3.0 washed 20 times

3. Unwashed PermaNet 2.0

4. PermaNet 2.0 washed 20 times

5. Polyester net, conventionally treated with deltamethrin at 25 mg/m^2^, washed until just before exhaustion

6. Untreated polyester net

Each net was deliberately holed with six 4 cm × 4 cm holes to simulate a worn net. The trial took place between 7 July and 4 October 2008. The treatment arms were rotated 3 times through the huts according to a Latin Square design. A treatment was assigned at random to a particular hut for 3 nights' observation before being rotated to the next hut. Male volunteers slept on beds under the net which were tucked under the mattress. The six sleepers were rotated through the six huts on consecutive nights. Data were collected for 54 nights. Three nets were available per treatment arm and each net was tested on consecutive nights during the three-night rotation. At the end of each rotation the huts were cleaned and aired for one day and the treatments moved to the next hut.

White sheets were laid over the veranda and room floors to ease the collection of knocked-down mosquitoes. Each morning after dawn, mosquitoes were collected using aspirators from the floor, walls, exit traps and inside the nets, scored as dead or alive and as fed or unfed and identified to species using a binocular microscope. Live mosquitoes were held for 24 h with sugar solution in paper cups to determine delayed mortality.

The primary outcomes were:

▪ deterrence - reduction in hut entry relative to the control huts fitted with untreated nets

▪ treatment induced exiting - the proportion of mosquitoes found in exit traps relative to control huts

▪ blood-feeding inhibition - the proportional reduction in blood feeding relative to untreated nets

▪ mortality - the proportion of mosquitoes killed

The first and third of these outcomes are indicators of personal protection which can be estimated by the equation:

% personal protection = 100(B_u _- B_t_)/B_u_

where B_u = _is the total number blood-fed in the huts with untreated nets, and B_t _is the total number blood-fed in the huts with treated nets.

The overall killing effect of the treatment was estimated by the equation:

Insecticidal effect (%) = 100(K_t _- K_u_)/T_u_

where K_t _is the number killed in the huts with treated nets, K_u _is the number dying in the huts with untreated nets, and T_u _is the total collected from the huts with untreated nets.

The criteria for approval was that the PermaNet 3.0 LN washed 20 times or more should perform according to these outcomes equal to or better than a conventionally treated net washed till just before exhaustion. Twenty washes is set by WHO as the average number of washes a LLIN is likely to incur during its life, assuming nets are washed 4 times a year and last up to 5 years.

#### Assessment of toxicity of nets used in the experimental hut trial

WHO cone bioassays were performed on a randomly selected net from each of the six treatment arms using laboratory reared *An. gambiae *Kisumu at three intervals: before any washing, after completion of the washing cycles, and after completion of the hut trial. Four pieces of netting measuring 30 cm × 30 cm were cut along a diagonal transect on the four side panels and a further piece was cut from the top panel. Three replicate bioassay tests were carried out on each side panel and 10 replicate tests on the top panel using five mosquitoes per replicate.

#### Chemical analysis of nets used in the experimental hut trial

Chemical analysis was conducted on PermaNet 2.0, PermaNet 3.0 and CTN from the 5 treatment arms before washing, after washing and after the hut trial. Taking one net per treatment arm, five 30 cm × 30 cm samples were cut from the four side panels and the one top panel of each net before and after washing and post hut trial. From each sample pieces were also taken for determination of density or homogenized and an analytical portion of 300 mg taken for determination of deltamethrin, deltamethrin R-isomer and/or PBO.

Deltamethrin, deltamethrin R-isomer and piperonyl butoxide were extracted by heating under reflux for 60 minutes with xylene and determined by gas chromatography with flame isonisation detection (GC-FID) using the internal standard calibration.

### Analysis

The analysis of experimental hut data were carried out using logistic regression for proportional data (proportions blood-feeding, dying and exiting each night) and negative binomial regression for numeric data (numbers collected, dying and feeding each night) after adjusting for the effects of individual huts and sleepers. Data was analysed using Stata 9 software (Stata Co., College Station, TX, USA).

Proportional data from laboratory bioassay tests (cone tests and tunnel tests) were normalised using arcsine square root transformation and the replicate test data analysed using analysis of variance [40].

### Ethical clearance

Approval was obtained from the ethics review committees of the London School of Hygiene and Tropical Medicine, the Tanzanian National Institute of Medical Research (Ref: NIMR/HQ/R.8a/Vol. X/86) and the World Health Organization. Each trial participant gave written informed and was offered chemoprophylaxis during and for one month after the experimental hut trial.

The procedure for use of guinea pigs in tunnel tests conformed with criteria established in EC Directive 86/609/ECC regarding protection of animals used for experimental purposes. The procedure accorded with published guidelines of the World Health Organization and was approved by the Tanzanian National Institute of Medical Research Project Review Committee.

## Results

### Exploratory bioassay tests on PermaNet 3.0, PermaNet 2.0 and CTN washed up to 20 times

#### Cone bioassays

**Figure 1:**
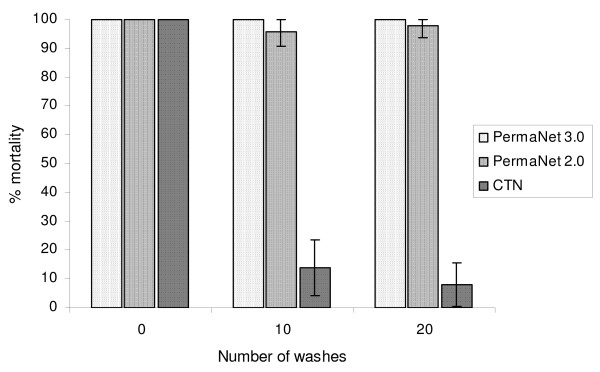
**Efficacy of treated netting after washing as determined by WHO cone bioassay tests with *Anopheles gambiae *Kisumu**.

After 20 washes PermaNet 3.0 induced 100% mortality, PermaNet 2.0 induced 98% mortality and the deltamethrin-treated CTN induced 8% mortality in *An. gambiae *Kisumu (Figure 1).

**Figure 2:**
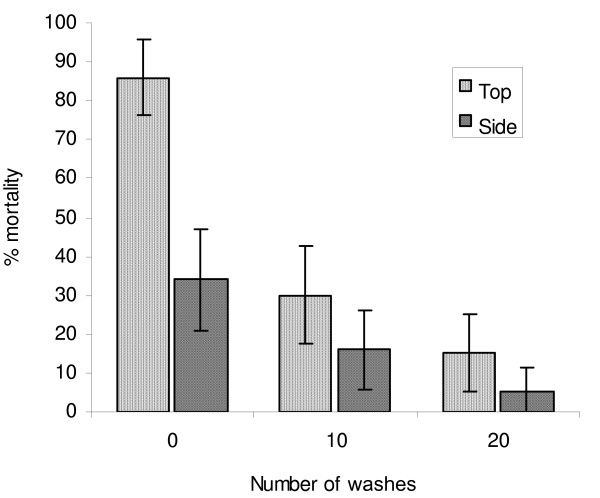
**Efficacy of PermaNet 3.0 top and side panels against pyrethroid resistant *Culex quinquefasciatus *Masimbani as determined by WHO cone bioassay tests**.

The percentage mortality induced by PermaNet 3.0 against the *Cx. quinquefasciatus *Masimbani pyrethroid resistant strain differed between roof and sides (Figure 2). The side netting induced only 33% mortality before washing, decreasing to 4% mortality after 20 washes. The roof netting induced higher rates of mortality than the sides: 86% at 0 washes, decreasing to 15% after 20 washes (P = 0.01). Thus the PBO was synergistic before washing but the effect was mostly lost between 10 and 20 washes (Figure 2).

### Tunnel tests

#### Passage

**Figure 3:**
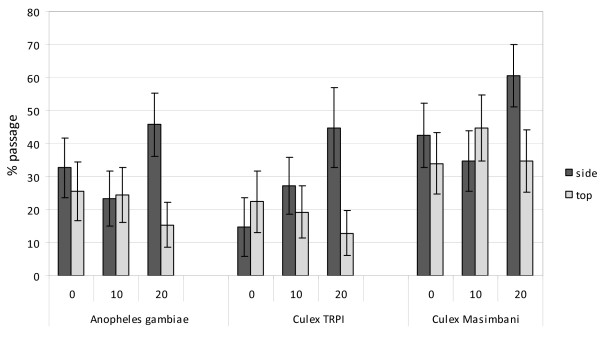
**Tunnel tests using PermaNet 3.0 top and side netting before and after washing (10 or 20 times) against pyrethroid susceptible (*Anopheles gambiae *and *Culex *TPRI) and resistant (*Culex *Masimbani) mosquitoes - Percentage passage through the holed netting**.

Each mosquito strain (*An. gambiae *Kisumu, *Cx. quinquefasciatus *TPRI and *Cx. quinquefasciatus *Masimbani) showed over 90% penetration through the untreated netting. All insecticide treatments inhibited passage of mosquitoes through the netting but less so for the pyrethroid resistant *Cx. quinquefasciatus *Masimbani (P = 0.014) (Figure 3). After 20 washes, passage of each strain was more inhibited through the PBO/deltamethin-treated top netting than through the deltamethrin-treated side netting (P = 0.04); passage through the PBO treated top netting was just as inhibited after 20 washes as at 0 washes (P = 0.45).

#### Blood feeding

**Figure 4:**
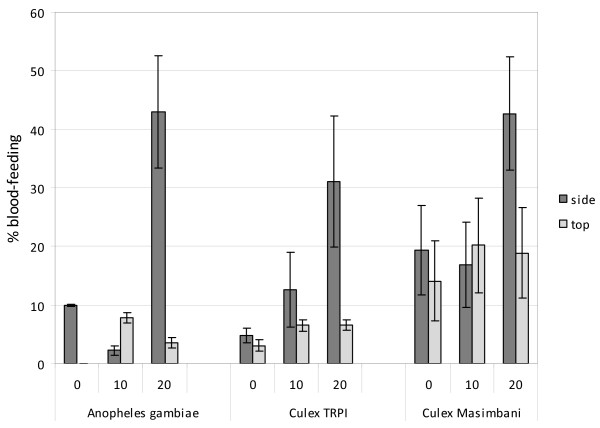
**Tunnel tests using PermaNet 3.0 top and side netting before and after washing (10 or 20 times) against pyrethroid susceptible (*Anopheles gambiae *and *Culex *TPRI) and resistant (*Culex *Masimbani) mosquitoes - Percentage blood-feeding**.

All three strains showed a high rate of feeding (78% or more) through the untreated netting. The blood-feeding trends for each of the treatments (Figure 4) mirrored that for passage trends (Figure 1a).

The *An. gambiae *Kisumu and *Cx. quinquefasciatus *TPRI susceptible strains showed low rates of feeding through the unwashed deltamethrin-treated side netting but higher rates of feeding through side netting washed 20 times (P = 0.07). The feeding rate associated with the PBO-deltamethrin impregnated netting was highly inhibited both in unwashed and in 20 washed samples.

The resistant *Cx. quinquefasciatus *Masimbani strain showed higher feeding rates than the susceptible *Cx. quinquefasciatus *TPRI strain (or *An. gambiae*) through treated netting at each wash interval (P = 0.01). The feeding rate of the resistant *Cx. quinquefasciatus *Masimbani was notably higher through the deltamethrin-treated side netting after 20 washes. The feeding rate observed with PBO/deltamethrin-impregnated top netting did not change significantly with washing (P = 0.34) and after 20 washes was only half that observed with the deltamethrin side netting.

**Figure 5:**
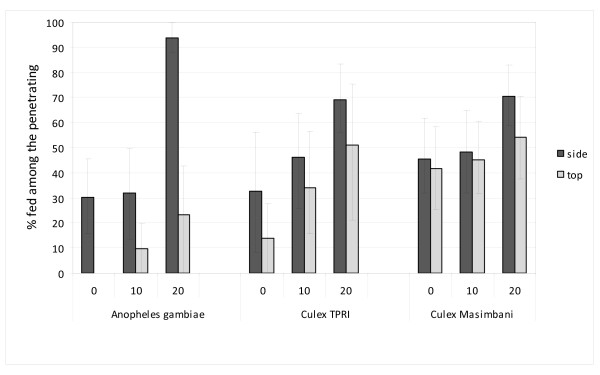
**Tunnel tests using PermaNet 3.0 top and side netting before and after washing (10 or 20 times) against pyrethroid susceptible (*Anopheles gambiae *and *Culex *TPRI) and resistant (*Culex *Masimbani) mosquitoes - Percentage blood-feeding among those mosquitoes that penetrated the netting**.

The trend in feeding rate among mosquitoes penetrating the holed netting showed a gradual increase over the course of 20 washes regardless of whether the netting was treated with deltamethrin or PBO-deltamethrin (Figure 5). For the two susceptible strains tested, the proportion feeding was always less with the PBO-deltamethrin netting than with the deltamethrin netting. The pyrethroid resistant *Cx. quinquefasciatus *Masimbani strain showed little or no difference in the proportion feeding between the deltamethin-treated and PBO/deltamethrin-treated netting.

#### Mortality

**Figure 6:**
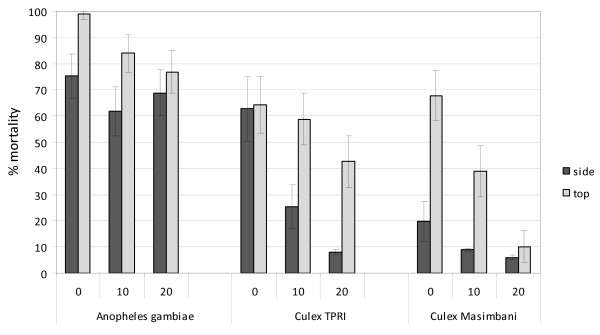
**Tunnel tests using PermaNet 3.0 top and side netting before and after washing (10 or 20 times) against pyrethroid susceptible (*Anopheles gambiae *and *Culex *TPRI) and resistant (*Culex *Masimbani) mosquitoes - Percentage mortality**.

The untreated nets recorded zero mortality against all three strains. Both types of treated netting induced greater mortality against *An. gambiae *Kisumu than against *Cx. quinquefasciatus *susceptible and resistant strains (P = 0.001) (Figure 6). The mortality rate against *An. gambiae *Kisumu was greater with PBO-deltamethrin netting than with deltamethrin netting (P = 0.04). Washing the two types of netting up to 20 times did not have a significant effect on mortality of the highly susceptible *An. gambiae *Kisumu (P = 0.21).

The pyrethroid susceptible *Cx. quinquefasciatus *TPRI strain recorded greater mortality than susceptible *An. gambiae *Kisumu on each type of netting at the corresponding wash interval (P = 0.01). The pyrethroid resistant *Cx. quinquefasciatus *Masimbani strain recorded greater mortality (20%) than the susceptible TPRI strain (62%) on unwashed deltamethrin-treated side netting (P = 0.047). Mortality rates in both strains decreased after washing, indicating removal of deltamethrin by the washing process. The difference in mortality between resistant and susceptible strains became progressively smaller with washing (17% difference at 10 washes) and was barely evident at 20 washes (2% difference). Hence the contact time of *Cx. quinquefasciatus *with the deltamethrin-treated net at 20 washes was insufficient to induce mortality in either strain.

The unwashed PBO-deltamethrin top netting induced an almost identical level of mortality in susceptible (64.2%) and resistant (67.7%) *Cx. quinquefasciatus*, indicating that PBO was synergizing the pyrethroid resistance in *Cx. quinquefasciatus *Masimbani. Over the course of 20 washes of the PBO-deltamethrin netting there was a partial loss of activity against the susceptible strain and a near complete loss of activity against the resistant strain. After 20 washes there was no significant difference in mortality induced against *Cx. quinquefasciatus *Masimbani by the top or side nettings (P = 0.68). This indicates that the surface concentration of PBO was largely removed by washing so no further synergy was evident against the resistant Masimbani strain and any PBO replenishment from the core of the fibres after washing was insufficient to regain toxic activity.

### Experimental hut trial

#### Determination of point of 'insecticide exhaustion'

The point of exhaustion is the number of washes at which cone bioassay mortality of *An. gambiae *Kisumu decreases to less than 80%. Mortality decreased below 80% after four washes, and hence CTNs washed three times were used as reference nets in the hut trials.

#### Resistance status

WHO resistance tests with deltamethrin 0.05% test papers on adults collected from huts at the start of the trial indicated that *An. gambiae *was susceptible (100% mortality) and *Cx. quinquefasciatus *was resistant (52% mortality).

#### Assessment of toxicity of nets used in the experimental hut trial

Before washing of the trial nets, the percentage knockdown and mortality of *An. gambiae *Kisumu were recorded as 100% for each treatment arm. After 20 washes, PermaNet 3.0 still scored 100% mortality whereas PermaNet 2.0 scored 96% mortality. At the end of the 36 day trial, mortality in PermaNet 3.0 and 2.0 arms stood at 96% or more and in the CTN washed to just before exhaustion mortality stood at 90%.

#### Number of mosquitoes collected in the experimental huts

*Anopheles gambiae *sensu strictu was the only member of the *An. gambiae *complex present at Zenet village. *An. gambiae *were more abundant than *Cx. quinquefasciatus *during the trial (Tables 1 and 2). The average number of *An. gambiae *per treatment ranged from 8 to 14 females per night. *Culex quinquefasciatus *ranged from 1 to 1.8 per night. Fewer *An. gambiae *females were collected from the huts with treated nets, but the difference compared to the untreated control was not significant except for the unwashed PermaNet 3.0 which showed 41% deterrence (P = 0.03).

**Table 1:** Experimental hut summary for *Anopheles gambiae*.

	Untreated net	PermaNet 3.0 Unwashed	PermaNet 2.0 Unwashed	PermaNet 3.0 washed 20 times	PermaNet 2.0 washed 20 times	CTN washed 3 times
**Total females caught**	**723**	**425**	**574**	**558**	**586**	**560**
Females caught per night	13^a^	8^b^	11^ab^	10^ab^	11^ab^	10^ab^
% Deterrence	-	41	21	23	19	22

**Total females in veranda and exit traps**	**618**	**335**	**491**	**474**	**518**	**515**
% Exiting (95% C.I.)	86^a ^(83-88)	79^b ^(75-82)	86^a ^(82-88)	85^a ^(82-88)	88^a ^(86-91)	92^c ^(89-943.9)

**Total females blood fed**	**202**	**11**	**59**	**58**	**54**	**59**
% Blood fed (95% C.I.)	28^a ^(25-31)	3^b ^(1-5)	10^c ^(8-13)	10^c ^(8-13)	9^c ^(7-12)	11^c ^(8-13)
% Blood feeding inhibition	-	91	63	63	67	63
% Personal protection	0^a^	95^b^	71^c^	71^c^	73^c^	71^c^

**Total females dead**	**108**	**407**	**548**	**531**	**510**	**411**
% Mortality (95% C.I.)	15^a ^(13-18)	96^b ^(93-97)	96^b ^(93-97)	95^b ^(93-97)	87^c ^(84-90)	73^d ^(70-77)
% Mortality corrected for control	-	95	95	94	85	69
% Overall killing effect	0^a^	41^b^	62^b^	59^b^	56^b^	42^b^

Numbers in the same row sharing a letter superscript do not differ significantly (P > 0.05).

**Table 2:** Experimental hut summary for *Culex quinquefasciatus*.

	Untreated net	PermaNet 3.0 Unwashed	PermaNet 2.0 Unwashed	PermaNet 3.0 washed 20 times	PermaNet 2.0 washed 20 times	CTN washed 3 times
**Total females caught**	**81**	**70**	**52**	**96**	**87**	**68**
Females caught per night	1.5^a^	1.3^a^	0.9^a^	1.8^a^	1.6^a^	1.3^a^
% Deterrence		14	36	0	0	16

**Total females in veranda and exit traps**	**46**	**65**	**51**	**95**	**84**	**61**
% Exiting (95% C.I.)	57^a ^(46-67)	93^bc ^(84-97)	98^bc ^(88-100)	99.0^c ^(93-100)	97^bc ^(90-99)	90^b ^(80-95)

**Total females blood fed**	**41**	**4**	**3**	**0**	**0**	**10**
% Blood fed (95% C.I.)	51^a ^(40-61)	6^bc ^(2-14)	6^bc ^(2-16)	0^c^	0^c^	15^b ^(8-25)
% Blood feeding inhibition	-	89	89	100	100	71
% Personal protection	0^a^	90^b^	93^b^	100^b^	100^b^	76^b^

**Total females dead**	**5**	**36**	**19**	**35**	**30**	**26**
% mortality (95% C.I.)	6^a ^(3-14)	51^b ^(40-63)	37^c ^(25-50)	37^c ^(28-46)	34^c ^(25-45)	38^c ^(28-50)
% Mortality corrected for control	-	48	32	32	30	34
% Overall killing effect	0^a^	38^b^	17^b^	37^b^	30^b^	26^b^

Numbers in the same row sharing a letter superscript do not differ significantly (P > 0.05).

#### Exiting rates

The proportion collected each morning from the veranda and window traps of huts with untreated nets was higher for *An. gambiae *(85.5%) than for *Cx. quinquefasciatus *(56.8%). Insecticide induced exiting from huts with treated nets relative to the huts with untreated nets was evident for *Cx. quinquefasciatus *but not for *An. gambiae*, because most of the latter exited naturally each night from the huts with untreated nets (Tables 1 and 2).

#### Blood-feeding

In huts with untreated nets, there was a higher rate of blood-feeding among *Cx. quinquefasciatus *than among *An. gambiae *(Tables 1 and 2). There was a significantly lower rate of blood-feeding among *Cx. quinquefasciatus *and *An. gambiae *in huts with treated nets (P = 0.0001).

*Anopheles gambiae *showed the lowest blood-feeding rate in huts with the unwashed PermaNet 3.0. However, there was no significant difference in the *An. gambiae *feeding rates between PermaNet 3.0 washed 20 times, PermaNet 2.0 washed 20 times, PermaNet 2.0 unwashed or CTN washed to just before exhaustion.

The feeding rates of *Cx. quinquefasciatus *did not differ in huts with unwashed PermaNet 3.0 or unwashed PermaNet 2.0 (P = 0.73). Feeding rates after 20 washes were 0% for both PermaNet 3.0 and PermaNet 2.0. Huts with CTN washed three times recorded a feeding rate of 15%, a significantly higher rate than that recorded for PermaNet 3.0 washed 20 times or PermaNet 2.0 washed 20 times.

The PermaNet 3.0 and PermaNet 2.0 washed 20 times, and the CTN washed three times, scored similar levels of personal protection against *An. gambiae *(71%, 73% and 71% respectively). The personal protection recorded against *Cx. quinquefasciatus *with the PermaNet 2.0, PermaNet 3.0 and CTN were not significantly different from one another.

#### Mortality

The huts with LLINs and CTNs recorded much greater levels of mortality of *An. gambiae *and *Cx. quinquefasciatus *than huts with untreated nets.

Against *An. gambiae *there was no difference between the proportions killed by the unwashed PermaNet 3.0 or unwashed PermaNet 2.0 (P = 0.79); both induced greater than 95% mortality. Against *Cx. quinquefasciatus*, the proportion killed by the unwashed PermaNet 3.0 (51%) was greater than the proportion killed by the unwashed PermaNet 2.0 (37%) (P = 0.05). The mortality associated with PermaNet 3.0 fell to 37% after 20 washes. The mortality associated with PermaNet 2.0 remained a consistent 36% and 35% at 0 and 20 washes respectively; both these values were virtually identical to the mortality induced by PermaNet 3.0 after 20 washes (37%) (P = 0.77). This means that any synergized toxicity by PBO in PermaNet 3.0 against *Cx. quinquefasciatus *was no longer evident after 20 washes. The mortality associated with PermaNet 3.0 and PermaNet 2.0 after 20 washes against *Cx. quinquefasciatus *was similar to that of the positive control (CTN washed three times) (P = 0.85) (Table 2).

Against *An. gambiae *the PermaNet 3.0 recorded higher mortality than PermaNet 2.0 after 20 washes (P = 0.001). The difference was small (8%) but may indicate that the PBO was not completely depleted but could still exert a limited effect against highly susceptible mosquitoes. Both PermaNet 3.0 and PermaNet 2.0 washed 20 times induced significantly greater mortality against *An. gambiae *than the conventionally treated net washed three times (P = 0.001).

The overall killing effect against *An. gambiae *(the proportion of mosquitoes killed by the treated nets relative to the number entering untreated huts) was similar in PermaNet 3.0 (59%) and PermaNet 2.0 (56%) when washed 20 times (P = 0.87). These overall killing effects were not significantly greater than that for the CTN washed three times (42%) (P = 0.89).

#### Chemical analysis

**Figure 7:**
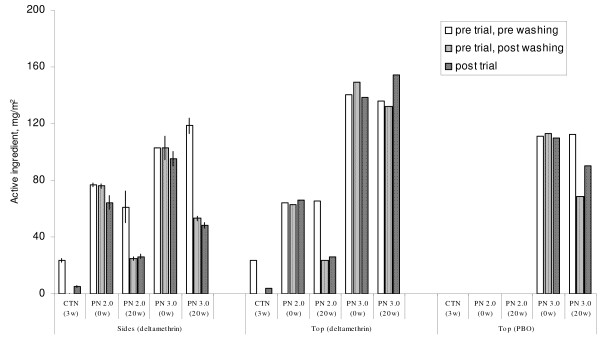
**Chemical analysis of deltamethrin and PBO content of PermaNets used in the hut trial**. Content of samples were analyzed pre and post washing and post trial. Number of washes are recorded in parenthesis on X axis. PBO content was 10 times the values recorded on the Y axis. Confidence intervals reported for deltamethrin on side netting.

The chemical analysis is presented in Figure 7. The loading dosage of deltamethrin on the polyester side panels of PermaNet 3.0 was recorded as 103 mg/m^2 ^in one sample and 119 mg/m^2 ^in a second sample. After 20 washes the quantity on the sides had decreased to 53 mg/m^2^. After the trial was completed the quantity had decreased still further to 28 mg/m^2^. The loading dosage of deltamethrin on the polyethylene top panel of PermaNet 3.0 was 136 mg/m^2^. After 20 washes it was recorded as 132 mg/m^2 ^and after the trial was completed it was recorded as 135 mg/m^2^. The lack of any evident decrease in content was presumably due to the deltamethrin on the top panel being incorporated or locked into the polyethylene fibre as opposed to being coated on the surface of the fibre on the polyester sides during manufacture.

The loading dose of deltamethrin in PermaNet 2.0 was 61 mg/m^2 ^in one sample and 77 mg/m^2 ^in another. After 20 washes the dose had decreased to 25 mg/m^2^; no further loss was recorded at the completion of the trial.

The PBO on the top panel of PermaNet 3.0 was 1142 mg/m^2 ^before washing, 684 mg/m^2 ^after 20 washes, and 1013 mg/m^2 ^at the end of the trial. The variation partly reflects the limited number of samples taken for analysis (only one sample taken post-washing and one post-trial). There was no evidence of any real change in PBO content due to washing.

## Discussion

Laboratory tunnel tests with PermaNet 3.0 produced results consistent with experimental hut trials and help to explain the trends in blood feeding and mortality associated with PermaNet 3.0 and PermaNet 2.0 in the field. The tunnel tests demonstrated a synergistic interaction of PBO and deltamethrin on roof netting against susceptible *An. gambiae *and both susceptible and resistant *Cx. quinquefasciatus *relative to netting from side panels treated with deltamethrin alone. This synergy was manifested in higher mortality, reduced passage through the holes and reduced feeding rates with netting treated with PBO-deltamethrin. The synergy in tunnels against pyrethroid resistant *Cx. quinquefasciatus *was progressively lost over 10 washes and fully lost after 20 washes. Cone bioassays on resistant *Cx. quinquefasciatus *confirmed the loss of synergy over 20 washes.

In the experimental hut trial both PermaNet 2.0 and PermaNet 3.0 induced high rates of mortality against pyrethroid susceptible *An. gambiae *at 0 and 20 washes and both rates exceeded that of the CTN washed to just before cut off point. On the basis of this result PermaNet 3.0, like PermaNet 2.0 before it, warrants interim approval by WHO as a LLIN [41]. It was encouraging that the *An. gambiae *blood feeding rate associated with the zero washed PermaNet 3.0 was lower than with the zero washed PermaNet 2.0. After 20 washes, however, the feeding rates between PermaNet 3.0 and PermaNet 2.0 no longer differed, indicating a loss of activity under field conditions.

It was initially encouraging that the mortality rate of pyrethroid resistant *Cx. quinquefasciatus *in huts with zero washed PermaNet 3.0 was higher than that with zero washed PermaNet 2.0. This indicated the PBO in PermaNet 3.0 was exerting a partial synergism against *Cx. quinquefasciatus*. As per tunnel test results the synergism in the huts was fully lost after 20 washes. Blood feeding rates in *Cx. quinquefasciatus *in the huts did not differ between PermaNet 2.0 and 3.0 either in unwashed or 20 washed nets. According to Khayrandish & Wood [35], WHO used synergists and nerve recordings to explore the resistance in *Cx. quinquefasciatus *from this region, enhanced oxidases and a nerve insensitivity mechanism, probably *kdr*, are responsible for pyrethroid resistance.

The roof netting showed little change in chemical content (either in deltamethrin or in PBO) after twenty washes. Certainly any small change observed was not sufficient to explain the large difference in efficacy (mortality) between unwashed and 20 times washed PermaNet 3.0 in cones, tunnel tests or experimental huts. This indicates that deltamethrin, the PBO or both compounds are depleted from the surface of the fibre after 20 washes and fail to migrate sufficiently from the core to the surface to allow full regeneration. It would seem that PBO rather than deltamethrin is the compound that remains locked in the fibre. The evidence for this stems from the zero difference in mortality in the tunnel tests between the 20 times washed PermaNet 3.0 and the 20 times washed PermaNet 2.0 against resistant Cx. quinquefasciatus taken together with the higher mortality with 20 times washed PermaNet 3.0 against susceptible *Cx. quinquefasciatus *(S): deltamethrin must still be present on the surface of both PermaNet 2.0 and 3.0 and causing some mortality of susceptible *Cx. quinquefasciatus *but there seems little or no PBO left on the surface of PermaNet 3.0 netting to allow synergy in resistant *Cx. quinquefasciatus*.

It is possible insufficient time was given between washing and tunnel testing for regeneration of PBO to occur. However, the evidence from the hut trial indicates this is not the reason since over the six weeks in the huts the PermaNet 3.0 washed 20 times showed no difference in performance to the PermaNet 2.0 washed 20 times, but during this six week interval there was plenty of time for the PBO to migrate to the surface of fibres. The no difference in *Cx. quinquefasciatus *mortality between PermaNet 3.0 washed 20 times and PermaNet 2.0 washed 20 times suggests a failure to regenerate.

It is possible that the higher mortality initially seen with PermaNet 3.0 relative to PermaNet 2.0 is due more to the higher loading dose of deltamethrin than to any contribution of PBO. An appropriate control to test this hypothesis - a 'PermaNet 3.0' loaded with the same dosage of deltamethrin but containing no PBO - was not available for testing. Such a control should always be considered in future testing of combination nets.

At present there is limited evidence that mosquitoes contact the roof of the net while seeking access to the host. This may not hold for all species, and more corroborative observations are required. Unless the majority of mosquitoes respond to host odour or convection plumes in this way, the 2-in-1 concept as a tactic for managing resistance management tactic is flawed. The higher mortality of *Culex *in huts with unwashed PermaNet 3.0 versus unwashed PermaNet 2.0 does, however, provide some support to the concept.

It is important to note that the laboratory tests and Phase II trials reported here refer to efficacy before and after standardized washing rather than to performance under long term household use. There is limited temporal dimension to this work because the interval between the start of washing and the completion of the trials was only three months. Because pyrethroids used on nets have low vapour pressure a pyrethroid LLIN that showed high efficacy after 20 Phase II washes might, quite reasonably, be expected to remain efficacious for at least three years of household use, as reported recently for PermaNet 2.0 and Olyset LLINs [41,42]. We have no information on how long the PBO component of PermaNet 3.0 would remain effective in the field as the synergist incorporated into netting may have different physical characteristics to pyrethroids. By contrast there is reasonable expectation on the basis of current knowledge that the pyrethroid in conventional LLIN would last for three years or more [37].

The negligible difference in mortality between PermaNet 3.0 and 2.0 against *An. gambiae *in huts either before or after washing would seem unlikely to provide additional control of *An. gambiae *populations; besides, mortality with PermaNet 2.0 was already very high. With pyrethroid resistant *Cx. quinquefasciatus *almost half survived exposure to PermaNet 3.0 in the huts and this proportion increased to 64% after washing. As a combination net designed to control pyrethroid resistance mediated by mixed function oxidase mechanisms the capacity of PermaNet 3.0 to control pyrethroid multiple resistant mosquitoes or prevent selection of resistance appears limited.

## Competing interests

The authors declare that they have no competing interests.

## Authors' contributions

PT carried out the hut trials, supervised the laboratory tests, analysed the data and drafted the report. SM supervised the field and laboratory trials and revised the report and manuscript. CM supervised the field trials. DM carried out the field trials. WS and JM carried out the laboratory tests. OP carried out the chemical analysis. MR analysed and interpreted the data and wrote the paper. All authors read and approved the final manuscript.

## Authors' information

Information on the Pan-African Malaria Vector Research Consortium can be found at the following website: http://www.pamverc.org.
